# Author Correction: Gene expression and functional deficits underlie TREM2-knockout microglia responses in human models of Alzheimer’s disease

**DOI:** 10.1038/s41467-023-36930-1

**Published:** 2023-03-02

**Authors:** Amanda McQuade, You Jung Kang, Jonathan Hasselmann, Amit Jairaman, Alexandra Sotelo, Morgan Coburn, Sepideh Kiani Shabestari, Jean Paul Chadarevian, Gianna Fote, Christina H. Tu, Emma Danhash, Jorge Silva, Eric Martinez, Carl Cotman, G. Aleph Prieto, Leslie M. Thompson, Joan S. Steffan, Ian Smith, Hayk Davtyan, Michael Cahalan, Hansang Cho, Mathew Blurton-Jones

**Affiliations:** 1 Department of Neurobiology & Behavior, University of California Irvine, Irvine, CA 92697 USA; 2 Sue and Bill Gross Stem Cell Research Center, University of California Irvine, Irvine, CA 92697 USA; 3 Institute for Memory Impairments and Neurological Disorders, University of California Irvine, Irvine, CA 92697 USA; 4 Department of Mechanical Engineering and Engineering Science, University of North Carolina Charlotte, Charlotte, NC 28223 USA; 5 Department of Biological Sciences, University of North Carolina Charlotte, Charlotte, NC 28223 USA; 6 Nanoscale Science Program, University of North Carolina Charlotte, Charlotte, NC 28223 USA; 7 Center for Biomedical Engineering and Science, University of North Carolina Charlotte, Charlotte, NC 28223 USA; 8 Department of Physiology and Biophysics, University of California Irvine, Irvine, CA 92697 USA; 9 Institute of Neurobiology, National Autonomous University of Mexico, Queretaro, Mexico; 10 Department of Psychology and Human Behavior, University of California Irvine, Irvine, CA 92697 USA; 11 Department of Biophysics, Sungkyunkwan University, Suwon, 16419 Korea

**Keywords:** Mechanisms of disease, Transcriptomics, Alzheimer's disease, Microglia

Correction to: *Nature Communications* 10.1038/s41467-020-19227-5, published online 23 October 2020

The original version of this article contained an error in the labelling of the bar graphs to the right of Fig. 3b, 3c, 3d and 3e, in which the symbols + and – representing addition or absence of the SYK inhibitor R406, respectively, were inadvertently switched. This has been corrected in the PDF and HTML versions of the article.

The original version of this article also contained an error in the title of the legend for Fig. 2. which incorrectly read ‘TREM2 knockout microglia exhibit decreased caspase activation at baseline and after cytokine starvation’. The correct version states ‘increased’ in place of ‘decreased’. This has been corrected in both the PDF and HTML versions of the article.

