# Supplementary material for: Gene expression and functional deficits underlie TREM2-knockout microglia responses in human models of Alzheimer’s disease
Source: Nat Commun. 2020 Oct 23;11:5370. doi: 10.1038/s41467-020-19227-5 (PMC7584603; doi:10.1038/s41467-020-19227-5)
Supplement: Supplementary file 1 — Supplementary Information [file 41467_2020_19227_MOESM1_ESM.pdf]

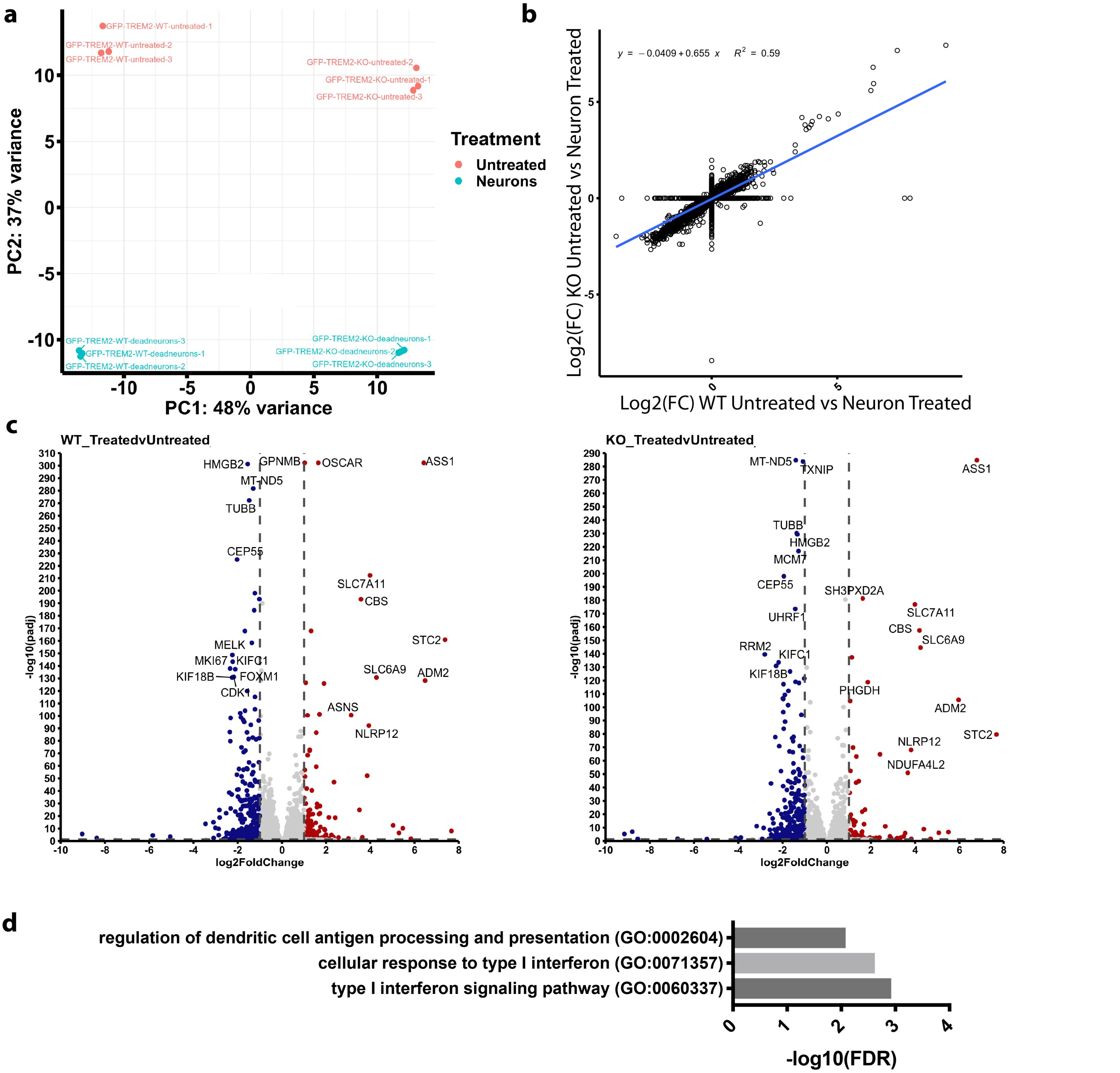

Supplemental Figure 1. Neuron treatment of isogenic microglia. **a** In iPS-microglia treated with dead neurons, both WT and TREM2 KO lines respond with similar magnitudes. The first 2 principal components (PC) are split on WT vs KO and treated verses untreated. Note that TREM2 knockout lines appear to respond with equal magnitude as their WT counterparts. **b** Linear regression of WT cells treated with neurons versus KO cells treated with neurons reveals alignment of the transcriptome response R squared= 0.59. **c** Volcano plots of differentially expressed genes between untreated vs treated TREM2 WT cells (left) and TREM2 KO cells (right) show majorly the same gene changes. **d** Recriprocally changed genes between knockout and treatment with neurons show only three significantly altered gene ontology groups.

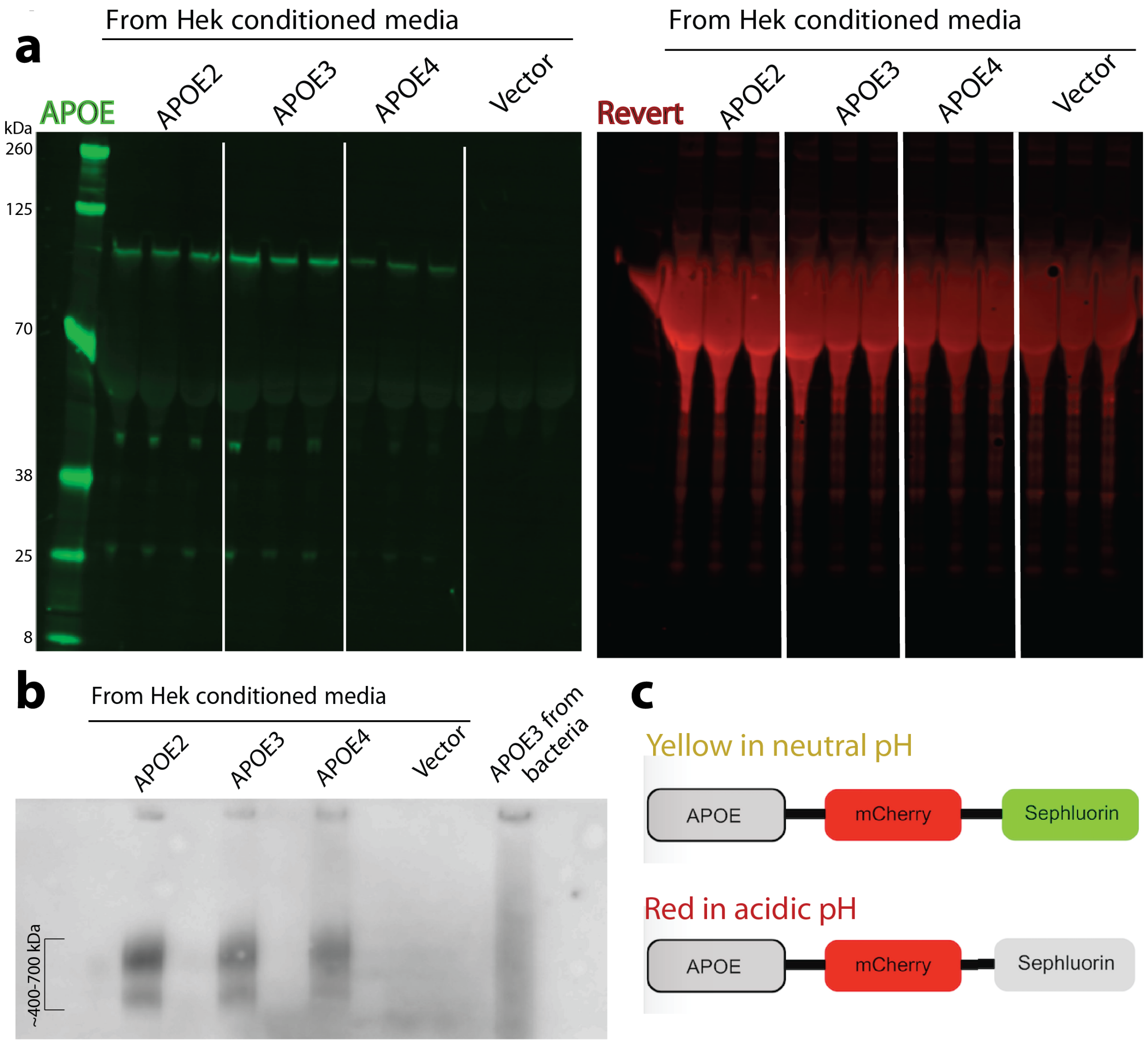

Supplementary Figure 2. APOE from HEK293T cells. Conditioned medium from transfected HEK293T cells was isolated and analyzed by (A) western blot (APOE-green; Revert total protein dye-red) and (B) native gel (Biolegend D6E10). Consistently, APOE4 is secreted at lower levels than APOE2&3 as has been published previously. Microgram amounts of APOE were normalized for experimentation. By native gel, APOE shows the expected double band which suggests it is properly lipidated. Non-lipidated APOE from bacteria is shown as a smear (negative control). (C) Schematic of construct used. In neutral pH, both mCherry and SEpHluorin are expressed simultaneously resulting in yellow color. In acidic pH, SEpHluorin is quenched and only red signal remains.

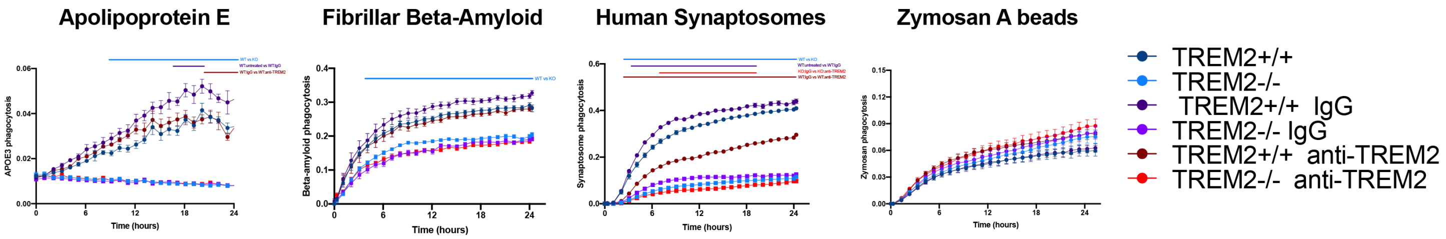

Supplemental Figure 3. Phagocytosis of all substrates after pre-treatement with anti-TREM2 antibody. TREM2 WT (dark colors) and KO (light colors) were pre-treated with 20 ng/mL IgG (purple) or anti-TREM2 (AF1828, red) for 15 min before the addition of fluorescent substrates. Bars above data representsignificance $p < 0.001$ from 2-way ANOVA with multiple comparisons at each time point.
Data are represented as mean values +/- SEM. (n=4 images each from 4 independent wells, experiments were replicated in all three isogenic backgrounds).

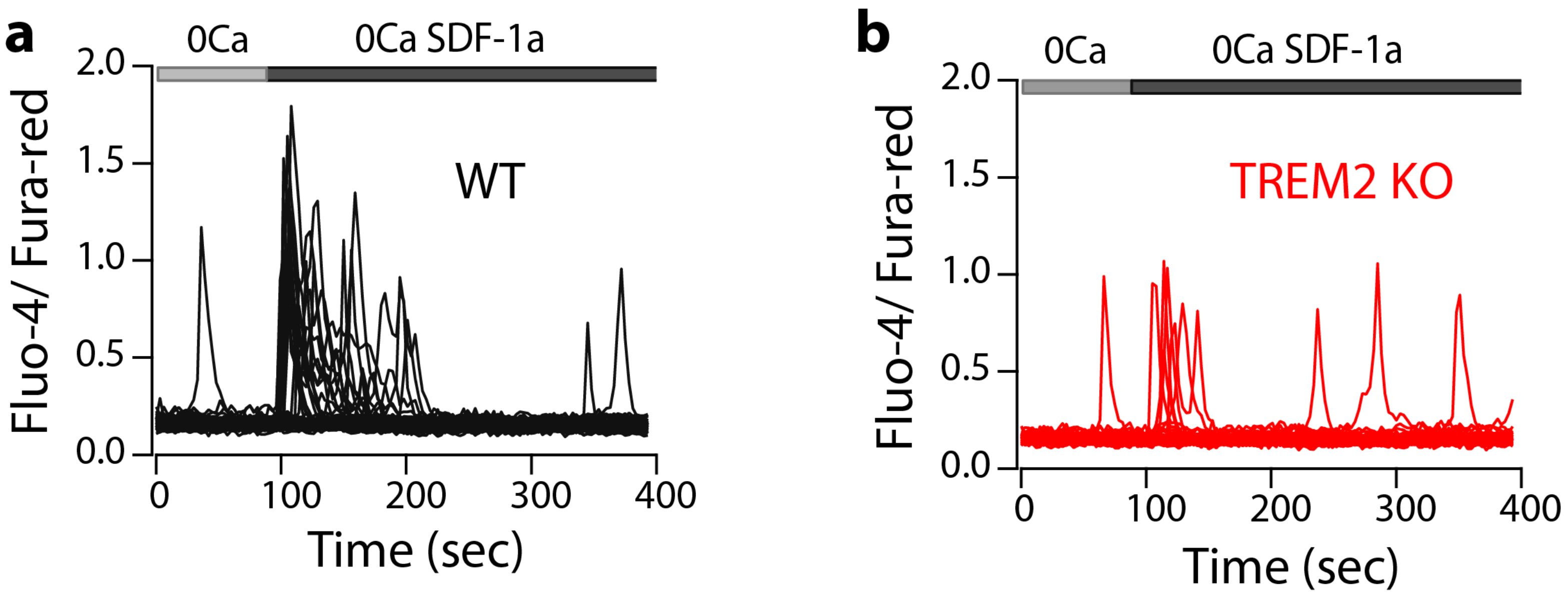

Supplemental Figure 4. Individual- cell traces of ratiometric calcium responses to SD-1a. Representative single-cell traces showing responses to SDF-1α in **a** WT and **b** TREM-2 KO microglia. Cells were imaged with Fluo-4 and Fura-Red. N= 111- 116 cells, 2 runs.

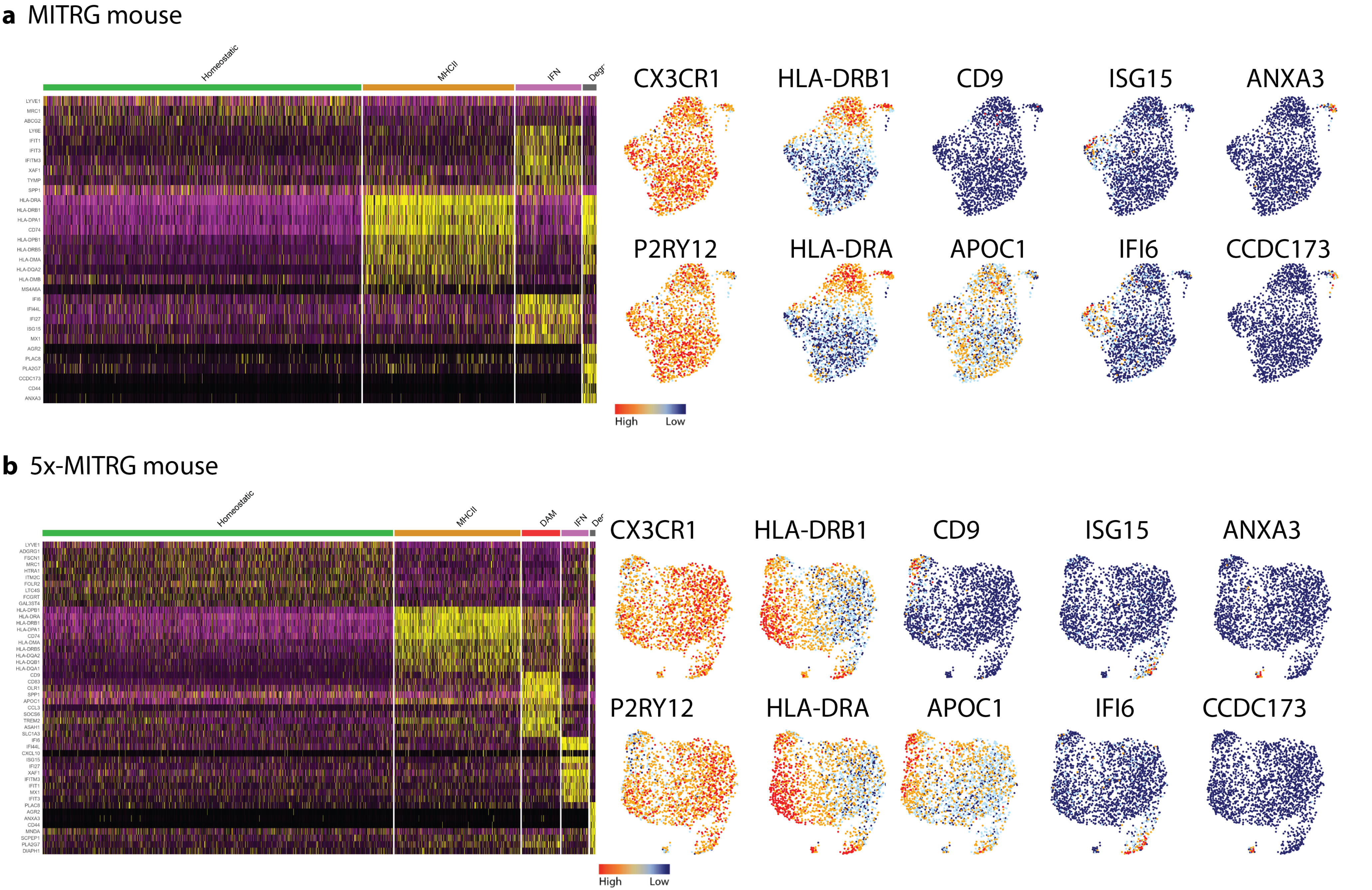

Supplemental Figure 5. Clustering and key gene expression for single-cell sequencing. Heatmaps for **a** MITRG and **b** 5x-MITRG mouse highlight key genes driving differences in clusters. These genes were used to define the naming scheme for each cluster. UMAP expression plots of key genes in each cluster are shown to the left. CX3CR1 and P2RY12 are high for homeostatic clusters. HLA-DRB1 and HLA-DRA are highly expressed in the HLA (MHCII) cluster. CD9 and APOC1 are expressed in DAMs. ISG15 and IFI6 are key interferon regulatory genes which are found in the interferon cluster. ANXA3 and CCDC173 demarcate the degranulaiton cluster.

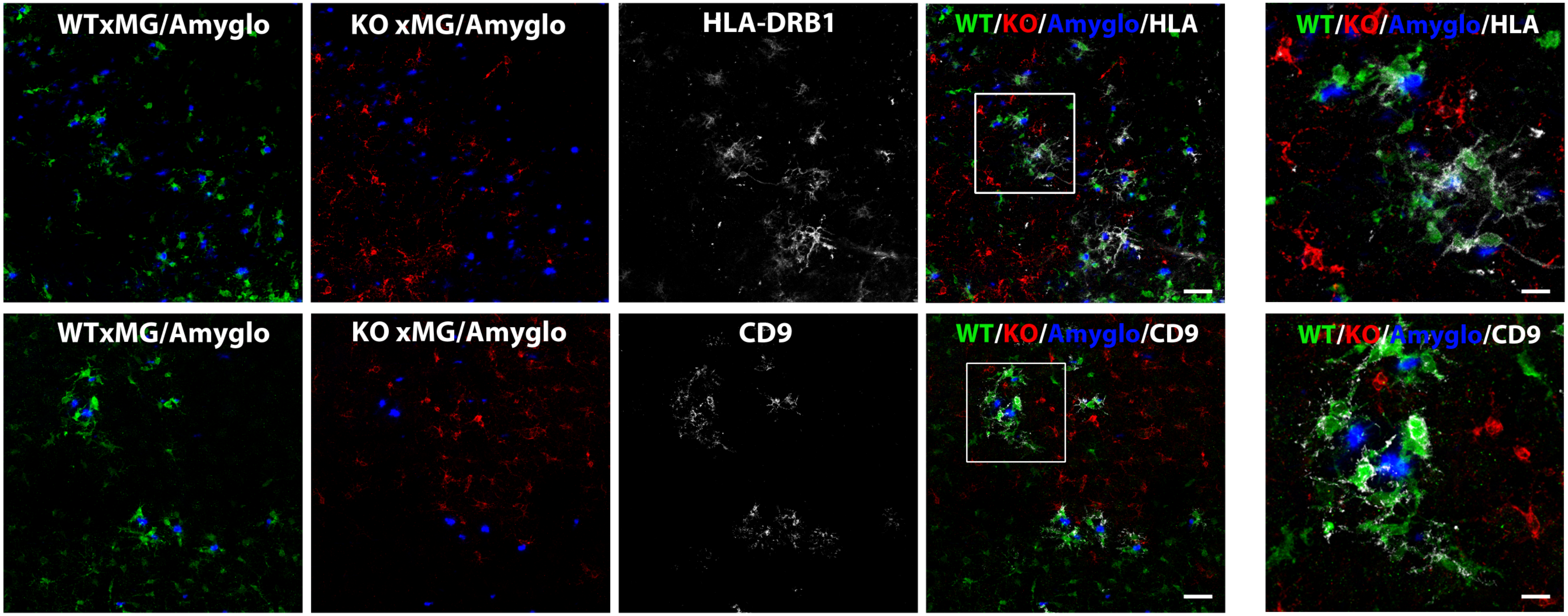

Supplemental Figure 6. CD9 and HLA expression in GFP -WT and RFP-KO lines. Histological analysis of human microglia within the 5x-MITRG mouse confirms TREM2 WT microglia (GFP+) express higher levels of the activation markers HLA-DRB1 (top) and CD9 (bottom) than TREM2 knockout microglia (RFP+). Scale bar = 40 μm for low power images and 10 μm for the high power images .

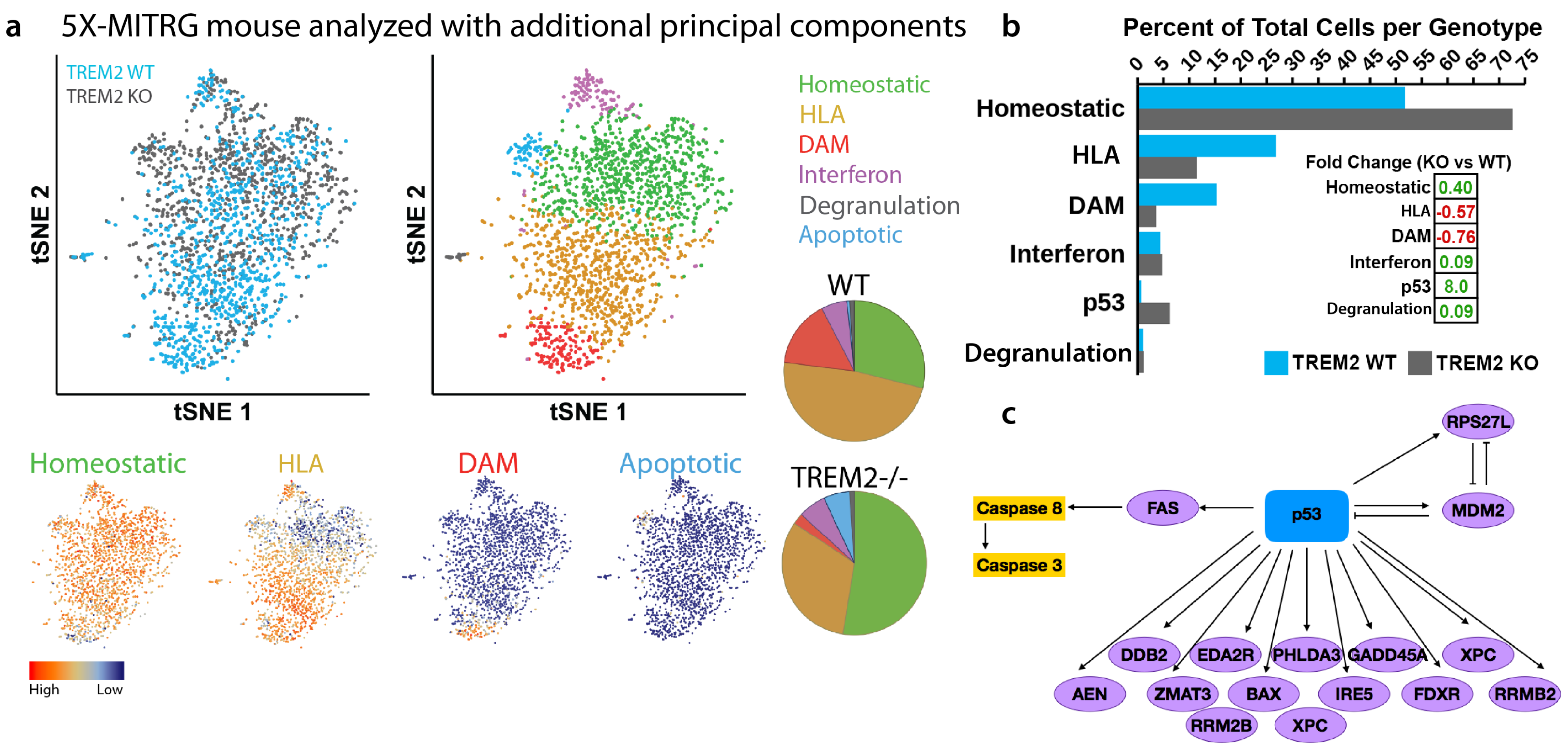

Supplemental Figure 7. Appearance of apoptotic cluster in sequencing analysis. **a** Analysis of single-cell sequencing data with additonal principal components reveals apoptotic cluster. **b** Same data as shown in pie charts in **a** visualized as bar graph and fold-change table Green values are increased in WT cells. Red values are decreased in WT cells compared to TREM2-/- cells. **c** Schematic of genes significantly enriched in the apoptotic cluster (purple) support this cluster as being p53 dependent.

Supplementary_Table_1_ClusterStatistcs_Seurat

	Homeostatic	MHCII/HLA	DAM	IFN	Degranulation
F_5X_WT_Cells	510	334	136	50	9
F_5X_WT_Clust_Percent	35.4	64.9	86.1	45.5	39.1
F_5X_WT_Percent_Total	49.1	32.1	13.1	4.8	0.9
F_5X_TR_Cells	929	181	22	60	14
F_5X_TR_Clust_Percent	64.6	35.1	13.9	54.5	60.9
F_5X_TR_Percent_Total	77	15	1.8	5	1.2
KOvsWT_Percentage_Fold	1.57	-2.14	-7.28	1.04	1.33

Rows	Homeostatic	MHCII/HLA	IFN	Degranulation
F_WT_WT_Cells	373	240	76	13
F_WT_WT_Clust_Percent	35.1	47.4	34.7	28.3
F_WT_WT_Percent_Total	53.1	34.2	10.8	1.9
F_WT_TR_Cells	691	266	143	33
F_WT_TR_Clust_Percent	64.9	52.6	65.3	71.7
F_WT_TR_Percent_Total	61	23.5	12.6	2.9
KOvsWT_Percentage_Fold	1.15	-1.46	1.17	1.53

Supplementary Table 2 Primer pairs

Generation of mouse models:
CSF1-Mut-F - TAA CTC CCA GCC TTC CAC AC
CSF1-Mut-R - GAC AAG CTC TGG GGA TTG AA
CSF2-Mut-F - CTT CTT CAG AAC CCC TTG GA
CSF2-Mut-R - CAA AGG TGG CTG CTT TAA GTG
Thpo-Mut-F - CAG GAC TGA AAA GGG AAT CA
Thpo-Mut-R - CGT TGG AAG GCC TTG AAT TT
Rag2-Mut-F - AGG TCC AAG CTG CTG CCA C
Rag2-Mut-R - GTG TGG GAT ATA GCT GTT GG
Il2rg-Com-F - CCA GAG AAA GAA GAG CAA GCA
Il2rg-Mut-R - GAT CCA GAT TGC CAA GGT GA
5x-PS1-F – AAT AGA GAA CGG CAG GAG CA
5x-PS1-R – GCC ATG AGG GCA CTA ATC AT

Generation of cell models:
Trem2_F: 5’ ctgcagagcaagcaagagtg
Trem2_R: 5’ ctggtagagacccgcatcat
