# Supplementary material for: Gene expression and functional deficits underlie TREM2-knockout microglia responses in human models of Alzheimer’s disease
Source: Nat Commun. 2020 Oct 23;11:5370. doi: 10.1038/s41467-020-19227-5 (PMC7584603; doi:10.1038/s41467-020-19227-5)
Supplement: Supplementary file 3 — Description of Additional Supplementary Files [file 41467_2020_19227_MOESM3_ESM.docx]

Supplemental Data 1 WT vs KO bulk RNA sequencing: RNA sequencing results comparing TREM2 WT and KO iPS-microglia. Differential expression analysis performed with DESeq2 as described in methods. Data was collected from 2 independent lines with 4 technical replicates. This file includes the average expression value of all data points (baseMean), log2(fold change) (log2FoldChange) and adjusted probability values from DESeq2 (padj). Raw data is available on GEO.

Supplemental Data 2 WT vs KO bulk RNA sequencing Neuron Treatment: RNA sequencing results comparing TREM2 WT and KO iPS-microglia treated with dead neurons. For 24 hrs Data was collected from one independent isogenic set with 4 technical replicates. This file includes the average expression value of all data points (baseMean), log2(fold change) (log2FoldChange) and adjusted probability values from DESeq2 (padj). Raw data is available on GEO.

Supplemental Data 3 WT vs KO bulk RNA sequencing Antibody Treatment: RNA sequencing results comparing TREM2 WT and KO iPS-microglia treated with IgG or anti-TREM2 antibody for 24 hrs. Data was collected from one independent line with 4 technical replicates. Differential expression analysis was performed with Genialis as detailed in the methods section. This file includes log2(fold change) (log_fc_value) and adjusted probability values from DESeq2 (prob_value). Raw data is available on GEO.

Supplemental Data 4 scRNA sequencing: Cluster Barcodes for single-cell sequencing. Cell barcodes for each cell genotype (TREM2WT or TREM2KO) in each mouse model (WT (MITRG) or 5X (5x-MITRG) are shown in the first two tabs.

Supplemental Data 5 Cluster Statistics from Combined Analysis: Single cell RNA sequencing analysis is described in the methods. Here, we present clustering statistics and to show percentages of each genotype (WT or TR (TREM2 KO)) within each mouse model (WT (MITRG) or 5X (5x-MITRG)). Cluster representation is shown both as a raw number of cells, (Cells), a percentage of the total cells in that cluster with both genotypes (Clust_Percent), and a percentage of the total cells of that genotype in any cluster (Percent_Total). In the differentially expressed genes tab, we show log2(fold change) (ave_logFC) and adjusted p-values (p_val_adj) which represent the comparison of differentially expressed genes from the cluster denoted on the right versus all other clusters. We also highlight the percentage of cells within the cluster denoted on the right which express the gene denoted on the first column (pct.1) as well as the percentage of cells in all clusters which express the gene denoted on the first column (pct.2). Raw data is available on GEO.
