# Supplementary material for: Gene expression and functional deficits underlie TREM2-knockout microglia responses in human models of Alzheimer’s disease
Source: Nat Commun. 2020 Oct 23;11:5370. doi: 10.1038/s41467-020-19227-5 (PMC7584603; doi:10.1038/s41467-020-19227-5)
Supplement: Supplementary file 11 — Reporting Summary [file 41467_2020_19227_MOESM11_ESM.pdf]

nature research

Corresponding author(s): Mathew Blurton-Jones

Last updated by author(s): Sep 4, 2020

Reporting Summary

Nature Research wishes to improve the reproducibility of the work that we publish. This form provides structure for consistency and transparency in reporting. For further information on Nature Research policies, see our Editorial Policies and the Editorial Policy Checklist.

Statistics

For all statistical analyses, confirm that the following items are present in the figure legend, table legend, main text, or Methods section.

n/a	Confirmed	
☐	☒	The exact sample size (*n*) for each experimental group/condition, given as a discrete number and unit of measurement
☐	☒	A statement on whether measurements were taken from distinct samples or whether the same sample was measured repeatedly
☐	☒	The statistical test(s) used AND whether they are one- or two-sided<br>*Only common tests should be described solely by name; describe more complex techniques in the Methods section.*
☒	☐	A description of all covariates tested
☐	☒	A description of any assumptions or corrections, such as tests of normality and adjustment for multiple comparisons
☐	☒	A full description of the statistical parameters including central tendency (e.g. means) or other basic estimates (e.g. regression coefficient) AND variation (e.g. standard deviation) or associated estimates of uncertainty (e.g. confidence intervals)
☐	☒	For null hypothesis testing, the test statistic (e.g. *F*, *t*, *r*) with confidence intervals, effect sizes, degrees of freedom and *P* value noted<br>*Give P values as exact values whenever suitable.*
☒	☐	For Bayesian analysis, information on the choice of priors and Markov chain Monte Carlo settings
☒	☐	For hierarchical and complex designs, identification of the appropriate level for tests and full reporting of outcomes
☒	☐	Estimates of effect sizes (e.g. Cohen's *d*, Pearson's *r*), indicating how they were calculated

Our web collection on statistics for biologists contains articles on many of the points above.

Software and code

Policy information about availability of computer code

Data collection	scRNA-seq library preparation was performed according to the 10X Chromium Single Cell 3' Reagents kit v3 user guide and loading the full sample volume onto the Chromium chip (20 uL containing ~15,000 cells). The workflow was then followed according to the 10X protocol and samples were pooled at equimolar concentrations for sequencing on an Illumina HiSeq 4000. FASTQ files were aligned to both the human GRCh38.p12 transcriptome (Ensembl release 95) and the mouse mm10 transcriptome (Ensembl release 95)67 using the CellRanger V3 count command, with the expected cells set to 5,000 and no secondary analysis performed. Following alignment, all reads that aligned to the mouse transcriptome were removed from the dataset before additional processing.
Data analysis	Software used has been referenced alongside the methods which use them. Analyses were conducted in: ImageJ 2.0.0, GraphPad Prism 7, ggplot2, FlowJo10.7.1, FACSDiva 9.0, and Olympus Cellsens 2.3<br><br>Bulk RNA-sequencing analysis. RNA-sequencing data was processed and interpreted using either the Genialis visual informatics platform or in-house bioinformatic analysis pipelines. RNA-sequencing read integrity was verified using FastQC. BBDuk was used to trim adapters and filter out poor quality reads. Remaining reads were then mapped to the hg19 reference genome using the HISAT aligner or the GRCh38 reference genome (Ensembl release 9767) using Kallisto v0.46.068. Complete clustering linkage was measured by pearson coefficient. Lowly expressed genes (expression count summed over all samples < 10) were removed before differential expression analysis. Differentially expressed genes were calculated using DESeq269 by applying FDR cutoffs between 0.01 and 0.001 and log fold change of at least 0.5. Geneset enrichment analysis was performed using EnrichR GO Biological Processes 2018. Volcano plots were generated using the 'ggplot2' R package70 and heatmaps were generated using the 'pheatmap' R package71.<br><br>Single Cell RNA Data Visualization and Differential Gene Analysis. UMI count tables were read into Seurat (v3)72 for preprocessing and clustering analysis. Initial QC was performed by log normalizing and scaling (default settings) each dataset followed by PCA performed using all genes in the dataset. Seurat's ElbowPlot function was used to select principal components (PCs) to be used for clustering along with a resolution parameter of 0.5 and clusters identified as being doublets, gene poor, or dividing were removed from the dataset prior to

downstream analysis. Secondary QC cutoffs were then applied to retain only cells with less than 30% ribosomal genes, 15% mitochondrial genes, greater than 600 genes but less than double the median gene count, and greater than 500 UMI but less than double the median UMI count.
Cells passing QC were then merged according to mouse of origin (e.g. TREM2 WT and KO microglia from the same 5x-MITRG mouse) using Seurat's 'merge' function and data were processed using Seurat's wrapper for the 'sctransform' R package73, while regressing out library size differences, percent ribosomal genes, and percent mitochondrial genes. PCA was then performed using the top 1,000 variable genes after removing ribosomal and mitochondrial genes from the lists (WT: 930 genes; 5X: 968 genes). For xMGs isolated from MITRG mice, a shared nearest neighbor (SNN) plot was generated using Seurat's 'FindNeighbors' function using PCs 1:20 as input, clustering was performed using the 'FindClusters' function and a resolution parameter of 0.5, and dimension reduction was performed using the 'RunUMAP' function with the same PCs used for generating the SNN plot. xMGs isolated from the 5X-MITRG mouse were processed similarly, using PCs 1:14 and a resolution parameter of 0.3. Differentially expressed genes were determined between clusters using the 'FindAllMarkers' function, which employs a Wilcoxon Rank Sum Test, with and FDR cutoff of 0.01, an LFC cutoff of 0.25, and the requirement that the gene be expressed in at least 10% of the cluster.
Information regarding the QC cutoffs and clustering parameters used in this analysis can be found in Supplemental Table 5.

scRNA-seq Differential Proportion Analysis. Cluster proportion tables for each sample were determined for both the MITRG and 5x-MITRG scRNA-seq datasets (Figure 6 a,b; Supplemental Table 5). Differential proportion analysis was then performed using the source code and techniques described by Farbehi et al. (PMID: 30912746). In short, a null distribution was generated for each cluster using the generateNULL() function with the number of permutations (n) set to 100,000 and the proportion of cells to be sampled (p) ranging from 0.05 to 0.3 by increments of 0.05. Actual cluster proportions were then compared to the null distributions and p-values were generated using the two.class.test() function.
Optimal parameters were determined by performing DPA on the clusters in MITRG mice and selecting the proportion parameter where no significance was observed for any cluster at a p-value less than or equal to 0.05 (Supplemental Table 5). The proportion value of 0.15 and a p-value cutoff of 0.05 was then applied to the 5x-MITRG analysis.

For manuscripts utilizing custom algorithms or software that are central to the research but not yet described in published literature, software must be made available to editors and reviewers. We strongly encourage code deposition in a community repository (e.g. GitHub). See the Nature Research guidelines for submitting code & software for further information.

Data

Policy information about availability of data

All manuscripts must include a data availability statement. This statement should provide the following information, where applicable:

- Accession codes, unique identifiers, or web links for publicly available datasets
- A list of figures that have associated raw data
- A description of any restrictions on data availability

The datasets generated and analyzed in this study are available through GEO (bulk sequencing: GSE157652 https://www.ncbi.nlm.nih.gov/geo/query/acc.cgi?acc=GSE157652; single-cell sequencing: GSE158234 https://www.ncbi.nlm.nih.gov/geo/query/acc.cgi?acc=GSE158234). Source data are provided in a Source Data File for all western blots (Figure 1a, 1g, supplemental Figure 2). Flow cytometry gating schema are provided in Source Data File (Figure 5b, 6c). Primers are provided in Supplementary Table 7. Any additional data presented in this paper is available from the corresponding author upon request.

Field-specific reporting

Please select the one below that is the best fit for your research. If you are not sure, read the appropriate sections before making your selection.

☒ Life sciences ☐ Behavioural & social sciences ☐ Ecological, evolutionary & environmental sciences

For a reference copy of the document with all sections, see nature.com/documents/nr-reporting-summary-flat.pdf

Life sciences study design

All studies must disclose on these points even when the disclosure is negative.

Sample size	Power analysis based on previous pilot studies informed sample sizes used for mouse studies, phagocytosis studies, calcium studies, flow cytometry, and migration (unpublished data from our lab). For RNA-sequencing, we have validated all pipelines with n=4 sample size in Hasselmann et al. Neuron 2019.
Data exclusions	No data was excluded
Replication	When possible, three isogenic sets were used to replicate experiments. It is noted in the text when this was not possible due to endogenous GFP fluorescence in one of the lines. Data reproduction was successful in all lines. The extent of effect of phagocytosis alterations varied between lines due to variable WT baseline levels of phagocytosis. The median line is represented in the manuscript. In vivo transplantation experiments were not replicated, however, data was reproduced in small pilot experiments using the same patient background.
Randomization	Allocation of mice was done at random by a lab technician. For all in vitro experiments, separate wells of differentiation were chosen at random as well.
Blinding	Investigators were blinded for all immunofluorescent imaging and analysis. Investigators were blinded to the hypothesis during calcium imaging and migration experiments. Phagocytosis and cell survival experiments were analyzed using pre-established incucyte software pipelines and thus allow for no user-bias. Western blot, HTRF, and flow cytometry experiments were not blinded.

Reporting for specific materials, systems and methods

We require information from authors about some types of materials, experimental systems and methods used in many studies. Here, indicate whether each material, system or method listed is relevant to your study. If you are not sure if a list item applies to your research, read the appropriate section before selecting a response.

Materials & experimental systems

n/a	Involved in the study
☐	☒ Antibodies
☐	☒ Eukaryotic cell lines
☒	☐ Palaeontology and archaeology
☐	☒ Animals and other organisms
☐	☒ Human research participants
☒	☐ Clinical data
☒	☐ Dual use research of concern

Methods

n/a	Involved in the study
☒	☐ ChIP-seq
☐	☒ Flow cytometry
☒	☐ MRI-based neuroimaging

Antibodies

Antibodies used	anti-GFP (1:500; Millipore Ab16901), anti-TagRFP (1:10,000; Kerafast EMU113), anti-Ku80 (1:250; Abcam ab79220), mounted with Fluoromount-G (SouthernBiotech). Additional samples were stained in anti-CXCR4 (1:100; MAB172 Clone 44716, R&D Systems), anti-CD9 (1:200; 312102, Biolegend), anti-HLA-DRB1 (1:200; 14-9956-82, Invitrogen), Goat Anti-Mouse (Millipore, AP308P; 1:10,000) or - Rabbit (Millipore, 12-348; 1:10,000) HRP conjugate, GAPDH (47724 Sigma, 1:5,000), Anti-P-SYK Cell signaling 2710 (1:1,000) anti-SYK Cell Signaling 13198 (1:2,000) anti-GAPDH (47724 Santa Cruz 1:5,000) , HRP-conjugated secondary antibody (115-035-146 Jackson Immunoresearch, 1:10,000), anti-APOE (803301, Biolegend, 1:1000)
Validation	anti-TREM2 antibodies are validated within our dataset on knockout controls. anti-Ku-80 antibody is validated in our dataset by co-staining with human specific antibodies, anti-GFP was validated for ICC and western blot by Millipore (https://www.emdmillipore.com/US/en/product/Anti-Green-Fluorescent-Protein-Antibody,MM_NF-AB16901). These antibodies were also used in Hasselmann et al. Neuron 2019. anti-CXCR4 is validated by immunohistochemistry and flow cytometry with unpublished data from Dr. Matthew Inlay's lab, anti-CD9 anti-HLA-DRB1 were validated for immunofluorescence and flow cytometry by publication in Hasselmann et al. Neuron 2019. anti-TagRFP was validated in non-fixed tissue to enhance staining in RFP positive cells.<br>SYK and pSYK were validated by CellSignaling and used in 23 and 74 publications respectively (https://www.cellsignal.com/products/primary-antibodies/syk-d3z1e-xp-rabbit-mab/13198) (https://www.cellsignal.com/products/primary-antibodies/phospho-syk-tyr525-526-c87c1-rabbit-mab/2710). Anti-GAPDH was validated in Marsh et al. PNAS 2016. anti-APOE was validated by Biolegend for western blot (https://www.biolegend.com/ja-jp/products/purified-anti-apo-e-antibody-11264) and confirmed previously to overlap with fluorescent signal from HEK293-T cells by Dr. Joan Steffan's lab.

Eukaryotic cell lines

Policy information about cell lines

Cell line source(s)	Human iPSC cell lines were generated by the University of California, Irvine Alzheimer's Disease Research Center (UCI ADRC) Induced Pluripotent Stem Cell Core from subject fibroblasts under approved Institutional Review Boards (IRB) and human Stem Cell Research Oversight (hSCRO) committee protocols.<br><br>SCC008 ReNcell® VM Human Neural Progenitor Cell LIne (https://www.emdmillipore.com/US/en/product/ReNcell-VM-Human-Neural-Progenitor-Cell-LIne,MM_NF-SCC008) was differentiated with SCR131 Human iPSC Derived Neural Progenitor Kit(Human iPSC Derived Neural Progenitor Kit)<br><br>293T (ATCC® CRL-3216™) HEK293T cells (https://www.atcc.org/products/all/crl-3216.aspx)
Authentication	The core uses non-integrating Sendai virus to perform al reprogramming thereby avoiding any integration-induced effects. G-banding karyotyping is performed for all iPSC lines every 10 passages by WiCell cytogenetics and only lines with normal and stable karyotypes are used. To assess pluripotency, all lines are examined by Pluritest, which compares pluripotentcy and differentiation associated gene expression to a matrix of over 13,000 pluripotent cell lines. To avoid the potential selection of latent or hidden infections all iPSC culturing and microglia differentiation and experimentation is performed without the use of antibiotics.<br>For CRISPR knockout, we use Cas9 protein we can greatly reduce off-target effects as the protein is degraded more rapidly than plasmid or virally-delivered Cas9. However, off target effects can still occur in genes that exhibit a high degree of homology to the guide RNA. To reduce this possibility, guide RNAs are designed using off-target prediction tools including the CRISPR design tool that is publicly available from the Broad Institute. In addition, we examined promising clones via PCR and sequencing the loci of the top four predicted off-target sites to confirm that no off-targeting has occurred before pursuing the reported experiments.<br><br>Other cell lines were not authenticated.

Mycoplasma contamination	Cell lines are tested monthly for mycoplasma and were determined to be negative.
Commonly misidentified lines (See ICLAC register)	No commonly misidentified lines were used.

Animals and other organisms

Policy information about studies involving animals; ARRIVE guidelines recommended for reporting animal research

Laboratory animals	The MITRG mouse was purchased from Jackson Laboratories (stock #017711); this BALB/c/129 model includes two knockouts alleles, Rag2- (Rag2tm1.1Flv), γc- (Il2rgtm1.1Flv), and three humanized knock-in alleles, M-CSFh (Csf1tm1(CSF1)Flv), IL-3/GM-CSFh (Csf2/Il3tm1.1(CSF2,IL3)Flv), TPOh (Thpotm1.1(TPO)Flv). The related and parental M-CSFh mouse line was also purchased form Jackson Laboratories (stock # 017708) and contains Rag2 and Il2rg deletions and humanized M-CSFh. The 5xFAD-MITRG model was created by backcrossing the MITRG mouse with 5xFAD mice which overexpress co-integrated transgenes for Familial Alzheimer's Disease (FAD) mutant APP (Swedish, Florida, and London) and mutant FAD PS1 (M146L and L286V). Progeny of these pairings were then genotyped and backcrossed with MITRG mice to return the 5 MITRG genes to homozygosity and maintain the APP/PS1 transgenic loci in the hemizygous state, resulting in the 5xfAD-MITRG model (Rag2-; γc-; M-CSFh; IL-3/GM-CSFh; TPOh; Tg (APPSwFlLon,PSEN1*M146L*L286V)6799Vas). All mice were age and sex matched and group housed on a 12h/12h light/dark cycle with food and water ad libitum. Mice are housed with ambient temperature and humidity. Mice were cared for from postnatal day 1 to 6 months of age. All animals presented in the manuscript are 6 mo old male.
Wild animals	No wild animals used.
Field-collected samples	No field animals used.
Ethics oversight	All animal procedures were conducted in accordance with the guidelines set forth by the National Institutes of Health and the University of California, Irvine (UCI) Institutional Animal Care and Use Committee (IACUC: AUP-17-162) who approved the study.

Note that full information on the approval of the study protocol must also be provided in the manuscript.

Human research participants

Policy information about studies involving human research participants

Population characteristics	Only one patient sample was used. Age: 92, Male, end-stage AD, PMI: 5.5 hr
Recruitment	Participants enrolled at the UCI Alzheimer's Disease Research Center (ADRC) are eligible for autopsy study. AD Patients from the ADRC core. (https://www.mind.uci.edu/adrc/neuropathology-core/) (https://www.mind.uci.edu/research-studies/brain-donation/)
Ethics oversight	Ethics oversight was approved independently by the Alzheimer's Disease Research Center (ADRC) at the University of California, Irvine (UCI).

Note that full information on the approval of the study protocol must also be provided in the manuscript.

Flow Cytometry

Plots

Confirm that:

- [x] The axis labels state the marker and fluorochrome used (e.g. CD4-FITC).
- [x] The axis scales are clearly visible. Include numbers along axes only for bottom left plot of group (a 'group' is an analysis of identical markers).
- [x] All plots are contour plots with outliers or pseudocolor plots.
- [x] A numerical value for number of cells or percentage (with statistics) is provided.

Methodology

Sample preparation	Tissue dissociation flow cytometry. Following perfusion with ice cold 1X DPBS containing 5 µg/ml actinomycin D, half brains were dissected, and the cerebellum was removed. Tissue was briefly stored on ice in RPMI 1640 containing 5 µg/mL actinomycin D, 10 uM triptolide, and 27.1 ug/mL anisomycin until subsequent perfusions were completed. Tissue dissociation was then performed utilizing the Tumor Dissociation kit, human (Miltenyi) and the gentleMACS Octo Dissociator with Heaters (Miltenyi) according to manufacturer guidelines with modifications. Briefly, tissue was cut into ~1 mm3 pieces and placed into the C-tubes with the kit's enzymes, 5 µg/mL actinomycin D, 10 µM triptolide, and 27.1 µg/mL anisomycin and samples were dissociated using the preprogrammed protocol. Following enzymatic digestion, samples were strained through a 70 um filter and pelleted by centrifugation. Myelin and debris were removed by resuspending the pellet in 8mL 23% Percoll, overlaid with 2 mL of 1X DPBS, spinning at 400 x G for 25 minutes at 4°C, with acceleration and brake set to 0, and discarding the myelin band and supernatant.<br><br>Microglia were treated with FC block (1:100; BD Biosciences 553142) 5 min in FACS buffer (DPBS, 2% BSA, 50 µM EGTA) before adding live/dead stain (1:100; Biolegend 423113), CD9-APC (1:100; Biolegend 312108) or CXCR4-APC (1:50; Biolegend

306510) for 30 min at 4 C in the dark. Samples were washed 3X in FACS buffer. 100,000 events per sample were collected on BD LSRFortessa with FACSDiva and analyzed in FlowJo 10.7.1. Gates were drawn on fluorescence minus one (FMO) controls.

Instrument	BD LSRFortessa
Software	FACSDiva 9.0 software was used to collect the data. Data was analyzed in FlowJo v10
Cell population abundance	For in vitro samples, iPS-microglia are pure and abundance of CXCR4 is high >90% of cells. For in vivo sorted microglia, there is significant debris in the sample (~20% microglia cells) and CD9 expression is expectedly low ~5-10% of microglia).
Gating strategy	Gating is described in Source Data file. From FSC/SSC, microglia were positively selected. From FSC-A/FSC-H single cells were selected. From SSC-A/SSC-W side doublets were eliminated (positively select single cells). From SSC/DAPI (Hoeschst) or FSC/PE (PI) live cells were positively selected. For Figure 4b, cells were next gated on APC:CXCR4+ cells. For Figure 5c, cells were split by TREM2 genotype via GFP/RFP. GFP+/RFP- or GFP-/RFP+ cells were positively selected and visualized for CD9:APC.

☒ Tick this box to confirm that a figure exemplifying the gating strategy is provided in the Supplementary Information.
